# Evidence-based Medicine Questions Logged by Emergency Medicine Residents On Shift in Relation to American Board of Emergency Medicine Content Areas

**DOI:** 10.5811/westjem.52907

**Published:** 2026-05-13

**Authors:** Shreyas Kudrimoti, Max Needham, Jacob R. Albers, Jeffrey B. Brown, Estelle Cervantes, Phillip Sgobba, Ajay K. Varadhan, Dawn M. Yenser, Bryan G. Kane

**Affiliations:** * Lehigh Valley Health Network, Department of Emergency and Hospital Medicine, Allentown, Pennsylvania; † University of South Florida Morsani College of Medicine, Lehigh Valley Campus, Allentown, Pennsylvania

## Abstract

**Introduction:**

Evidence-based medicine (EBM) skills are fundamental to lifelong learning. These can be tracked the same way that procedural skills are tracked—via residency program logs. Review of the logs can inform faculty on the EBM activity of their trainees. An understanding of the topics residents query while on shift can provide insight into where they need further knowledge to provide optimal patient care. Our objective in this project was to categorize the relationship of the clinical questions posed by emergency medicine (EM) residents while working in the emergency department to the American Board of Emergency Medicine (ABEM) Model of Clinical Practice.

**Methods:**

We conducted this institutional review board-approved study (deemed exempt research) in a postgraduate year (PGY) 1–4 EM residency. A toxicology rotation and fellowship were established during the study period. Residents were required to submit three to five descriptions of EBM activity per 28-day EM rotation block into the program’s management software. We analyzed each complete log submitted from June 2013–May 2020 using the 2019 ABEM Model of Clinical Practice. The clinical questions posed were mapped to the ABEM Model for content, including sub-categories and acuity level. Demographic information in the logs allowed for analysis for ABEM’s pediatric and geriatric modifiers. The primary outcome measure was the number of clinical questions mapped to each section of the Model.

**Results:**

From June 2013–May 2020, 10,444 discrete completed logs were completed by 137 residents. “Procedures and Skills” (n = 1,110, 10.63%) and “Cardiovascular Disorders” (n = 991, 9.49%) were the most prevalent ABEM content areas. “Trauma” (n = 812, 7.77%) and “Drugs and Chemical Classes” (n = 749, 7.17%) were the most prevalent ABEM sub-categories. “Emergent” (n = 7,770, 74.3%) was the most commonly searched ABEM acuity, followed by “lower acuity” (n = 5,341, 51.1%) and “critical” (n = 5,192, 49.7%). Of note, not all conditions have ABEM acuity codes, and some have multiple. Clinical questions addressed issues regarding pediatric patients in 10.16% (n = 1,061) and geriatric patients in 8.05% (n = 841) of logs.

**Conclusion:**

In this single-site cohort, “Procedures and Skills” was the most common source of on-shift questions for EM residents, perhaps representing just-in-time training. “Trauma” was the most common sub-category, potentially the result of a large footprint in the ABEM Model of Clinical Practice. The residency program’s toxicology rotation and fellowship may have influenced the types of conditions treated by residents and the subsequent content of their logs. Furthermore, completing logs on shift may have impacted the mapping to ABEM acuity levels. Programmatic understanding of residents’ on-shift, evidence-based medicine questions could serve to identify educational gaps and opportunities.

## INTRODUCTION

The modern idea and terminology of “evidence-based medicine” (EBM) was popularized by a 1992 announcement in the *Journal of the American Medical Association*.^1^ Evidence-based medicine represents a movement in medicine to use clinical research to directly guide clinical management.^1^ This EBM movement has given rise to advances in clinical practice that are commonplace today. As the principles of EBM became established, an instructional aspect of EBM emerged. The cornerstones of this teaching process were the essentials of “ask,” “acquire,” “appraise,” and “apply.”^2^ In its modern form EBM allows research and evidence to direct bedside care. Practicing medicine in this way can combine external clinical evidence, clinical expertise, and patient values.^3^ Ideally, the use of EBM in clinical practice works to consistently provide optimal, up-to-date care to patients.^4^ In real time while working in the emergency department (ED), the “ask,” “acquire,” “appraise,” and “apply” approach blends information-seeking and EBM.

The Accreditation Council for Graduate Medical Education acknowledges the importance of EBM, embedding it into program requirements.^5^ In emergency medicine (EM), EBM is measured via the practice-based learning (PBL) Milestone 1. Level 2 of PBL Milestone 1 is the ability of residents to “[articulate] the clinical questions … necessary to guide evidence-based care.”^6^ Although these EBM skills are commonly used across many hospital resident curricula, multicenter analysis of the use of EBM by EM residents has not been previously published. For example, Tavarez et al showed improved test scores on in-training examinations among EBM-trained pediatric fellows.^7^ Our group (Brown et al) has previously published research regarding the impact EBM may have on patient care.^8^ Extending that work, we sought to describe the clinical content of EBM questions asked by EM residents while on shift in the ED. Our goal in this study was to describe the relationship of EBM questions to the American Board of Emergency Medicine (ABEM) Model of Clinical Practice of EM. To do so, we mapped the content of the questions recorded in the residents’ logs of on-shift EBM activity to the 2019 ABEM Model.^9^

## METHODS

We conducted this study at a postgraduate year (PGY) 1–4 EM residency in an independent academic center sponsored by an integrated healthcare network based in eastern Pennsylvania. The study was reviewed by the network’s institutional review board, which deemed it exempt research. Within the network, residents rotated at two urban ED sites. One was located at a tertiary-care Level I trauma center with a dedicated pediatric ED, pediatric intensive care unit (ICU), and neonatal ICU. The other was a community ED at an institution with an inpatient psychiatric hospital. A toxicology fellowship with an associated toxicology rotation was developed during the study period. As described in Brown et al, the Program Evaluation Committee required that residents log on-shift EBM activity, referred to as PBL logs.^8^ Residents were required to complete three to five PBL logs (depending on the academic year) per 28-day EM rotation. The number of annual EM rotations varied from six for PGY-1 residents to eight for PGY-4 residents. Therefore, the total number of PBL logs a resident was required to complete in a given academic year ranged from 18–40. Logs were maintained in the residency program’s software management system New Innovations (New Innovations, Inc., Uniontown, OH).

**Population Health Research Capsule**

What do we already know about this issue?

*Evidence-based medicine (EBM) skills are fundamental to lifelong learning*.

What was the research question?

*How do the EBM questions asked by on-shift emergency medicine residents relate to the American Board of Emergency Medicine Model of Clinical Practice?*

What was the major finding of the study? *Residents asked about “Procedures and Skills” most frequently and “Environmental Disorders” least frequently*.

How does this improve population health?

*Understanding the content areas of residents’ on-shift EBM questions could serve to identify educational gaps and opportunities to improve patient care*.

The logs were based on a format posted to the listserv of the Council of Residency Directors in Emergency Medicine.^10^ The logs included a patient description, chief concern, discharge diagnosis, ED or hospital course, the clinical question the resident investigated, the search strategy used to investigate the question, and the information found regarding the question. Additionally, residents were required to report in the log the subsequent clinical application of the information they found by responding to the prompt, “Based on your research, would you have done anything differently?” These clinical applications of the information found by the residents are described in detail in the 2024 study by Brown et al.^8^

The EBM curriculum, described in the 2024 paper, occurred monthly and was embedded in the required weekly didactics.^8^ Expectations for the PBL logs were graduated, with a focus on the clinical question for PGY-1 residents based on the PICO format (Population/Patient/Problem, Intervention, Comparison, and Outcome). No expectations as to the clinical content were either defined or provided to the residents. When submitted, all logs were reviewed by a single member of the faculty to provide individualized feedback to the residents during their semi-annual evaluations. That feedback did not include review of content or topic but was limited to EBM skills, as noted in our prior paper.^8^ To be included in this analysis, logs needed to have been placed into New Innovations between June 2013–May 2020. Logs were anonymized to PGY and sex to be included in the research dataset, in accordance with Hadley et al.^11^

The logs were mapped to the topics noted in the 2019 ABEM Model of Clinical Practice.^9^ The mapping process was similar to previously described descriptive methodology.^12^ The Model has content areas, such as “Signs, Symptoms, and Presentations,” with subsequent categories and sub-categories, such as “Abnormal Vital Signs.” Each sub-category has one to three acuity levels: “critical”; “emergent”; and “lower acuity,” which are defined in the Model.^8^ “Critical” presentation indicates a high chance of mortality without immediate intervention; “emergent” indicates a likely progression in severity or complication without prompt treatment; and “lower acuity” suggests a low probability of the patient’s condition escalating. The definitions for pediatrics and geriatrics as special populations came from information provided by ABEM when describing the qualifying examination. The mapping was performed by three coders. These coders worked together on the first 200 logs to create a consensus for coding. They then met to discuss any logs in which coding was unclear. A single faculty member adjudicated any remaining coding questions. There was no formal measure of inter-rater reliability.^8^ We analyzed the results descriptively.

## RESULTS

From June 2013–May 2020, 11,145 total logs were entered into New Innovations, completed by 137 residents. Forty-eight residents (37%) were female. Of these logs, 10,444 were identified as complete and discrete (non-duplicates) and were used for this analysis. Table 1 demonstrates log proportion for each of the 20 ABEM content areas. The numbering aligns with the Model of Clinical Practice. Content area 19, “Procedures and Skills Integral to the Practice of EM,” which had the greatest number of logs with 1,110 (10.63%) entries, followed closely by “Cardiovascular Disorders” with 991 logs (9.49%). Infrequently queried on shift were “Environmental Disorders” (n = 142, 1.36%) and “Psychobehavioral Disorders” (n = 143, 1.37%). Table 2 demonstrates the 20 most frequently queried topic areas from the 2019 Model, from the most to least frequent. Given the level of detail contained within the ABEM Model, Table 2 provides a more granular sense of the clinical categories of EBM questions that EM residents decided to record in their logs.

“Emergent” (n = 7,770) was the most commonly searched ABEM acuity, followed by “lower acuity” (n = 5,341) and “critical” (n = 5,192). Of note, not all conditions have ABEM acuity codes, and some ABEM categories have multiple acuity codes. If an ABEM sub-category had all three levels of acuity, that log contributed to each acuity category. Therefore, the number of acuity categories is greater than the number of logs analyzed. In 1,061 logs (10.16%), the clinical question related to pediatric patients and in 841 (8.05%) to geriatric patients.

## DISCUSSION

The data reported from this study represent a broad picture of the types of patient presentations EBM activity was applied to at a single training program. The 10,444 individual logs demonstrate which content areas the EM residents most frequently investigated while on shift. In this study we attempted to define the first step in the “ask,” “acquire,” “appraise,” and “apply” approach to EBM by mapping the questions asked to the ABEM Model of Clinical Practice. In this cohort, “Procedures and Skills,” “Cardiovascular Disorders,” and “Abdominal and GI Disorders” were all present in relatively high proportions in the patient logs. The first, “Procedures and Skills,” may be explained by the use of just-in-time training for low-volume procedures. The high number of logs for cardiovascular and abdominal disorders may reflect the types of patients evaluated by the residents. A recent study noted that among non-trauma patients, abdominal pain was the most frequent concern, followed by dyspnea, fever, and chest pain.^13^

Prior work has demonstrated that 87% of EBM questions asked by residents arise when the trainee is not physically with the patient.^14^ Since the PBL logs included patient information such as medical record number, chief concern, and case outcome, the cohort should be focused on patient encounters rather than an educational topic that arose on shift, even if the questions arose outside the patient’s room. These examples of content areas may inform the need for EBM resources at training sites. For example, having easily accessible and curated procedural training resources may be of value. High-frequency topic areas could serve to inform residency leadership about gaps in the program’s didactics, as they represent areas of resident uncertainty.

At the other end, “Psychobehavioral Disorders,” “Environmental Disorders,” and “Hematologic and Oncologic Disorders” were less represented in the residents’ clinical questions. That psychiatric disorders were infrequently an area of residency inquiry is unlikely to reflect the proportion of patients they evaluated. The academic ED locations both contain dedicated behavioral sections, and one training ED is on the campus of one of the region’s large, inpatient psychiatric hospitals. In the case of behavioral emergencies, the volume of patients did not seem to impact the number of PBL logs placed. This inverse relationship may be the result of the single-site nature of the study but may also represent an opportunity for a program to identify content areas that require review by the program evaluation committee.

The type of patients about whom residents ask questions is interesting to note. The “emergent” ABEM acuity was the most highly represented acuity level in the dataset by a margin of more than 2,000 logs. The next most common acuity was “lower acuity,” with “critical” being the least common. In this sample, the residents did not opt to ask questions about critical patients as their first inclination. Given that the study could not control for logs placed later in a shift, or immediately after a shift while reflecting, lack of time to log a question during a resuscitation may not be the only reason why residents did not have as many queries in that category. The reason why critical patients were least likely to have a related PBL log requires further study, as the way in which EM residents engage in these PBL logs may provide insight into how emergency physicians behave as lifelong learners. If programs were to deploy PBL logs, the over-time trends in the topic areas of resident queries may inform programs, as discussed above.

## LIMITATIONS

This cohort is limited by its single-site nature. Additionally, the coding process, using a single faculty member to adjudicate the work of the primary reviewers, may have introduced bias. The impact of the internal EBM curriculum, the didactic curriculum provided, and the local patient population all likely affected the types of EBM queries the residents submitted. That the submissions included only a sample of the patients seen by the residents is another limitation; the residents may only have recorded a certain type of inquiry that they individually deemed appropriate for a PBL log. Another study limitation is that the only demographic information collected for residents was sex and PGY. It is possible that other demographic factors, such as age or prior experience, may have influenced the types of questions residents recorded in their logs. Additionally, residents may have been searching for “just-in-time information” rather than for best evidence/best practice. This limitation may be most pronounced when considering logs covering topics such as procedures.

Some observed trends may not be externally valid. For example, “Drugs and Chemical Classes” was among the most highly represented sub-categories present in the patient logs. Residents may have used EBM practices to ask questions about this competency because toxicology-related issues are rare, nuanced, and often high acuity. Put another way, the residents may have been accessing important “just-in-time” information. Another possible explanation of the high proportion of “Drugs and Chemical Classes”-related clinical questions may also indicate a special interest for this cohort of residents because of the strength of the local toxicology rotation, fellowship, and faculty. Therefore, should residency programs adopt an EBM activity like the PBL logs described here, local analysis, especially of the sub-categories, may be important. That the logs analyzed were > 10,000 in number, included each of the 20 ABEM major categories, and were collected over seven academic years, hopefully mitigates some of the limitations to the generalizability of the study.

## CONCLUSION

In this single-site cohort, the use of practice-based learning logs provided insight into the clinical content areas in which EM residents ask evidence-based medicine questions while on shift. Here, the “Procedures and Skills” category was the most common source of on-shift questions for EM residents, perhaps representing just-in-time training. “Trauma” was the most common sub-category, which may be the result of its large footprint in the ABEM Model of Clinical Practice. The residency program has a toxicology rotation and fellowship, which may have influenced these results observed. Furthermore, completing logs on shift may have impacted the mapping to ABEM acuity levels. Programmatic understanding of the content areas of resident on-shift EBM questions could serve to identify educational gaps and opportunities.

## Supplementary Information



## Figures and Tables

**Table 1:** Evidence-based medicine logs kept by emergency medicine residents while working in the emergency department, in a study categorizing the relationship of the clinical questions they asked to the American Board of Emergency Medicine Model of Clinical Practice.

ABEM content area	Number of logs	Proportion of total
1. Signs, Symptoms and Presentations	892	8.54%
2. Abdominal and GI Disorders	878	8.41%
3. Cardiovascular Disorders	991	9.49%
4. Cutaneous Disorders	263	2.52%
5. Endocrine, Metabolic, and Nutritional Disorders	292	2.8%
6. Environmental Disorders	142	1.36%
7. Head, Ear, Eye, Nose, Throat Disorders	559	5.35%
8. Hematologic and Oncologic Disorders	244	2.34%
9. Immune System Disorders	252	2.41%
10. Systemic Infection Disorders	472	4.52%
11. Musculoskeletal Disorders (Non-traumatic)	303	2.90%
12. Nervous System Disorders	801	7.67%
13. Obstetrics and Gynecology	356	3.41%
14. Psychobehavioral Disorders	143	1.37%
15. Renal and Urogenital Disorders	375	3.59%
16. Thoracic-Respiratory Disorders	737	7.06%
17. Toxicologic Disorders	748	7.17%
18. Traumatic Disorders	861	8.24%
19. Procedures and Skills Integral to Practice of EM	1110	10.63%
20. Other Core Competencies to Practice of EM	25	0.24%
Totals	10,444	100%

*ABEM*, American Board of Emergency Medicine; *EM*, emergency medicine; *GI*, gastrointestinal.

**Table 2:** Most common American Board of Emergency Medicine (ABEM) 2019 Model of Clinical Practice sub-categories identified in evidence-based medicine logs kept by residents while on shift, in a study categorizing the relationship of the clinical questions they asked in relation to the ABEM Model.

Rank	ABEM sub-category	Number of logs	Proportion of total
1	18.1 Trauma	812	7.77%
2	17.1 Drugs and Chemical Classes	749	7.17%
3	1.3 General	527	5.05%
4	19.4 Diagnostic and Therapeutic Procedures	500	4.79%
5	3.5 Diseases of the Myocardium, Acquired	316	3.03%
6	16.4 Obstructive/Restrictive Lung Disease	268	2.57%
7	1.2 Pain	266	2.55%
8	3.3 Disorders of Circulation	263	2.52%
9	2.9 Large Bowel	251	2.40%
10	3.4 Disturbances of Cardiac Rhythm	239	2.29%
11	7.4 Oropharynx/Throat	223	2.13%
12	10.6 Viral	220	2.11%
13	13.3 Complications of Pregnancy	218	2.09%
14	19.5 Ultrasound	182	1.74%
15	16.6 Pulmonary Embolism/Infarct	176	1.69%
16	4.4 Infections	168	1.61%
17	16.7 Pulmonary Infections	164	1.57%
18	2.7 Stomach	163	1.56%
19	19.2 Resuscitation	162	1.55%
20	9.2 Hypersensitivity	159	1.52%
20	19.3 Anesthesia and Acute Pain Management	159	1.52%

*ABEM*, American Board of Emergency Medicine.
