# Supplementary material for: Evidence-based Medicine Questions Logged by Emergency Medicine Residents On Shift in Relation to American Board of Emergency Medicine Content Areas
Source: West J Emerg Med. 2026 May 13;27(3):534–9. doi: 10.5811/westjem.52907 (PMC13246198; doi:10.5811/westjem.52907)
Supplement: Supplementary file 1 [file wjem-27-534-s001.docx]

Appendix 1: SRQR (Standards for Reporting Qualitative Research) Guidelines Checklist

| **No.** | **Topic** | **Item** | **Addressed in Paper** |
| --- | --- | --- | --- |
|  | **Title and abstract** |  |  |
| S1 | Title | Concise description of the nature and topic of the study identifying the study as qualitative or indicating the approach (e.g., ethnography, grounded theory) or data collection methods (e.g., interview, focus group) is recommended | Nature and topic of the study are included in the title, as well as the data collection method (on-shift evidence-based medicine questions). |
| S2 | Abstract | Summary of key elements of the study using the abstract format of the intended publication; typically includes background, purpose, methods, results, conclusions | Abstract is formatted to WestJEM guidelines. |
|  | **Introduction** |  |  |
| S3 | Problem formulation | Description and significance of the problem/phenomenon studied, review of relevant theory and empirical work; problem statement | Related past work and significance of studying resident on-shift EBM questions are described (third paragraph). Relevant theory on EBM and PBL logs (first and second paragraph) is described. Problem statement, that EM resident usage of EBM needs study, is given (lines 57-58). |
| S4 | Purpose or research question | Purpose of the study and specific objectives or questions | Purpose of the study, to describe the relationship of residents’ EBM questions to the ABEM Model, is stated (lines 62-5). |
|  | **Methods** |  |  |
| S5 | Qualitative approach and research paradigm | Qualitative approach (e.g., ethnography, grounded theory, case study, phenomenology, narrative research) and guiding theory if appropriate; identifying the research paradigm (e.g., postpositivist, constructivist/interpretivist) is also recommended; rationale | The Methods state that data were collected from resident self-reported practice based learning logs (lines 74-75). See lines 81-88 for a description of the logs’ content. |
| S6 | Researcher characteristics and reflexivity | Researchers’ characteristics that may influence the research, including personal attributes, qualifications/experience, relationship with participants, assumptions, and/or presuppositions; potential or actual interaction between researchers’ characteristics and the research questions, approach, methods, results, and/or transferability | The Methods state a residency faculty member reviewed all logs. The potential for bias was noted in the Limitations. |
| S7 | Context | Setting/site and salient contextual factors; rationale | The residency program’s location and characteristics are described (lines 67-74). |
| S8 | Sampling strategy | How and why research participants, documents, or events were selected; criteria for deciding when no further sampling was necessary (e.g., sampling saturation); rationale | The sample included logs entered between June 2013 and May 2020 (lines 97-98). Only logs that were complete and non-duplicates were included in the analysis (116-117). |
| S9 | Ethical issues pertaining to human subjects | Documentation of approval by an appropriate ethics review board and participant consent, or explanation for lack thereof; other confidentiality and data security issues | This study was approved by the Institutional Review Board (line 69-70). |
| S10 | Data collection methods | Types of data collected; details of data collection procedures including (as appropriate) start and stop dates of data collection and analysis, iterative process, triangulation of sources/methods, and modification of procedures in response to evolving study findings; rationale | The data collected in the logs (lines 81-88), the method of collecting logs in New Innovations (79-80), the dates of data collection (97-98), and the coding process (119-113) are all described. |
| S11 | Data collection instruments and technologies | Description of instruments (e.g., interview guides, questionnaires) and devices (e.g., audio recorders) used for data collection; if/how the instrument(s) changed over the course of the study | Logs were entered into the New Innovations software (lines 79-80). |
| S12 | Units of study | Number and relevant characteristics of participants, documents, or events included in the study; level of participation (could be reported in results) | Number and demographics of participants, number of entered logs, and number of analyzed logs are listed in lines 115-117. |
| S13 | Data processing | Methods for processing data prior to and during analysis, including transcription, data entry, data management and security, verification of data integrity, data coding, and anonymization/deidentification of excerpts | Anonymization of data (lines 98-99) and coding (109-113) are described. |
| S14 | Data analysis | Process by which inferences, themes, etc., were identified and developed, including the researchers involved in data analysis; usually references a specific paradigm or approach; rationale | The coding process is described (lines 109-113). |
| S15 | Techniques to enhance trustworthiness | Techniques to enhance trustworthiness and credibility of data analysis (e.g., member checking, audit trail, triangulation); rationale | A single faculty member adjudicated any coding questions (lines 111-112). |
|  | **Results/findings** |  |  |
| S16 | Synthesis and interpretation | Main findings (e.g., interpretations, inferences, and themes); might include development of a theory or model, or integration with prior research or theory | Most common content areas, sub-categories, and acuities associated with log entries are noted in the results, particularly Tables 1 and 2. |
| S17 | Links to empirical data | Evidence (e.g., quotes, field notes, text excerpts, photographs) to substantiate analytic findings | N/A. |
|  | **Discussion** |  |  |
| S18 | Integration with prior work, implications, transferability, and contribution(s) to the field | Short summary of main findings; explanation of how findings and conclusions connect to, support, elaborate on, or challenge conclusions of earlier scholarship; discussion of scope of application/generalizability; identification of unique contribution(s) to scholarship in a discipline or field | A short summary of which content areas were most common (lines 147-148), connections to the literature (152-157), limitations to generalizability (Limitations), and unique contributions (157-160) are given. |
| S19 | Limitations | Trustworthiness and limitations of findings | See Limitations. |
|  | **Other** |  |  |
| S20 | Conflicts of interest | Potential sources of influence or perceived influence on study conduct and conclusions; how these were managed | The authors declare no conflicts of interest. |
| S21 | Funding | Sources of funding and other support; role of funders in data collection, interpretation, and reporting | This study did not receive funding. |
